# Kinematics approach with neural networks for early detection of sepsis (KANNEDS)

**DOI:** 10.1186/s12911-021-01529-3

**Published:** 2021-05-20

**Authors:** Márcio Freire Cruz, Naoaki Ono, Ming Huang, Md. Altaf-Ul-Amin, Shigehiko Kanaya, Carlos Arthur Mattos Teixeira Cavalcante

**Affiliations:** 1 Graduate School of Science and Technology, Nara Institute of Science and Technology, Takayama, Ikoma, Nara 8916-5 Japan; 2 Graduate Program in Mechatronics, Federal University of Bahia, Salvador, Bahia 40170-110 Brazil; 3 Data Science Center, Nara Institute of Science and Technology, Takayama, Ikoma, Nara 8916-5 Japan

**Keywords:** Kinematics, Neural network, Sepsis, Early detection, Vital sign, Machine learning

## Abstract

**Background:**

Sepsis is a severe illness that affects millions of people worldwide, and its early detection is critical for effective treatment outcomes. In recent years, researchers have used models to classify positive patients or identify the probability for sepsis using vital signs and other time-series variables as input.

**Methods:**

In our study, we analyzed patients’ conditions by their kinematics position, velocity, and acceleration, in a six-dimensional space defined by six vital signs. The patient is affected by the disease after a period if the position gets “near” to a calculated sepsis position in space. We imputed these kinematics features as explanatory variables of long short-term memory (LSTM), convolutional neural network (CNN) and linear neural network (LNN) and compared the prediction accuracies with only the vital signs as input. The dataset used contained information of approximately 4800 patients, each with 48 hourly registers.

**Results:**

We demonstrated that the kinematics features models had an improved performance compared with vital signs models. The kinematics features model of LSTM achieved the best accuracy, 0.803, which was nine points higher than the vital signs model. Although with lesser accuracies, the kinematics features models of the CNN and LNN showed better performances than vital signs models.

**Conclusion:**

Applying our novel approach for early detection of sepsis using neural networks will prove to be an invaluable, more accurate method than considering only simple vital signs as input variables. We expect that other researchers with similar objectives can use the model presented in this innovative approach to improve their results.

## Background

Sepsis is a fatal organic dysfunction caused by a patient’s deregulated response to infection, and septic shock is a subset of sepsis where circulatory and cellular/metabolic dysfunction occurs with a higher risk of mortality [1, 2]. Sepsis is considered as the disease with the highest rate of death from infection [3]. Overall, the occurrence of sepsis and septic shock treated in a hospital is 437 and 270 cases per 100,000 person-years, respectively, with a total mortality rate of 26% [3, 4]. Nevertheless, according to Rhodes et al. [5], similar to most other diseases, early identification and management of the patient in the initial hours significantly improve the results of treatment.

Currently, several hospitals use the sepsis clinical score, called sequential organ failure assessment (SOFA), to define if a patient is diagnosed with sepsis. The score was recommended by the Third International Consensus Definitions for Sepsis and Septic Shock (Sepsis-3) [1]. The authors also proposed quickSOFA, or qSOFA, as the rapid score bedside criteria to facilitate the identification of patients with suspected infection and high risk of death [6]. The sepsis-3 criteria require monitoring patients’ vital signs (VS), such as heart rate, body temperature, blood pressure, and laboratory test results, among other variables, and calculating a sepsis score based on these values. Therefore, monitoring a patients’ variables is essential for the diagnosis and treatment as well as for medical analyses in studies that improve the medicine [7, 8].

In recent years, several studies have used VS data and other variables to predict sepsis and septic shock. Henry et al. [9] analyzed data from patients in ICUs and developed an algorithm based on survival analysis and supervised learning techniques to predict septic shock.

Manaktala and Claypool [10] proposed a system to detect sepsis and to provide decision support to medical staff during patient monitoring through a mobile app. The authors employed demographic, VS, medications, laboratory results, and nursing notes data to perform the detection and the decision support.

Horng et al. [11] showed—through tokenization algorithms, bigram detection, and text denial, in addition to machine learning—the possibilities of using data sourced from the text of patients’ medical registers, demographic data, and VS to identify patients with suspected infection and sepsis.

Mao et al. [12] developed a sepsis prediction algorithm based on a machine learning technique called gradient tree boosting, using six VS commonly available in ICUs.

Bock et al. [13] presented a new type of shapelet, a technique for identifying subsequences of time-series data that are statistically most significant for prediction. The authors showed shapelet patterns using VS as indicators of the severity of future sepsis.

Kamaleswaran et al. [14] compared the performances of some machine learning techniques to predict the onset of severe sepsis in children using VS as input.

Mohammed et al. [15] used five VS collected every minute and a support vector machine classifier to detect sepsis approximately 17 h before its onset.

Perng et al. [16] proposed a convolutional neural network (CNN) to predict sepsis-related mortality. They used 53 selected clinical variables and showed that the accuracy of the CNN model was higher than other machine learning methods and qSOFA.

Li et al. [17], Lin et al. [18], and Lauritsen et al. [19] proposed new model architectures composed especially of CNN and long-short term memory (LSTM) neural networks for predicting sepsis. The authors proved that their approach was more efficient for the early detection.

Lake et al. [20] studied 4096 intervals (25 min) of respiratory rates of neonatal ICU patients using approximate entropy [21] and sample entropy [22]. They verified that entropy values were lower right before the sepsis onset.

Ahmad et al. [23] analyzed the continuous heart rate of patients hospitalized for bone marrow transplantation and observed a 25% reduction in sample entropy before the diagnosis of sepsis.

Drewry et al. [24] proposed a temperature curve analysis that could find an abnormal pattern before sepsis onset, using the maximum, minimum, and variations of the ICU patients' temperature continuous time-series.

Additionally, Shashikumar et al. [25] analyzed the dynamics of continuous blood pressure and heart rate time-series to predict sepsis in ICU patients. They found that entropy-based measures of the two VS dynamics are independent predictors of sepsis.

Even though other important works have dealt with sepsis prediction in the past decade, we can consider those mentioned above a representative sample of the scientific community’s goals in this regard. These goals are, in most cases, to implement efficient models based on statistics, machine learning, and deep learning, to detect the most precise sepsis onset, to achieve the highest prediction accuracy as early as possible, to investigate the dynamics of measured features (variables) and to use the smallest number of easily collected features.

The above prediction models analyze the behavior of time-series variables until they classify the patient as positive for sepsis or identify a threshold probability for sepsis as early as possible. Although these studies have achieved commendable results, we propose a novel approach to accomplish some goals of the scientific community. The innovation is that we consider, at each timestamp, a “distance” measurement between the values of the patient’s variables and a proposed sepsis point. We developed a sepsis prediction model that considers not only the patient’s time-series VS behavior but also their kinematics over time, including position, velocity, and acceleration.

Therefore, we assumed that a patient can be represented by a point moving in an *n*-dimensional space, where *n* is the number of VS. We defined sepsis point as a single target point in this vital sign space. The position of the sepsis point is estimated from the patients’ VS values at their sepsis onset. Then, at each instant of time, we computed the relative distance between the patient and the sepsis point positions, as well as their relative velocity and acceleration. We named these variables as kinematics features (KF). When the KF were included as input variables of sepsis prediction neural networks (KF model), we verified that accuracies were higher compared to including only VS as input (VS model). In this work, we used a database where the sepsis-3 criteria were applied to define the sepsis onset of patients who developed the disease in the ICU.

We expect that other researchers with similar objectives can use this innovative approach to enhance their early detection models. To promote the reproducibility of our work and contribute to its scientific expansion, we published sample data and essential source code in [26].

## Methods

### Kinematics analysis of patient’s variables

#### Patient’s position, velocity, and acceleration

In our approach, we represented a patient as a point in an *n*-dimensional space, and the patient’s $n$ VS values at each instant of time are the coordinates that define the patient’s position vector [27, 28].

Thus, the VS values ${x}_{1 }, {x}_{2 },\dots ,{x}_{n}$ at time $t$ are the values of the components of the position vector ${\overrightarrow{r}}_{{A}_{i}}$ of a patient ${A}_{i}$ at time $t$, that is:

$${\overrightarrow{r}}_{{A}_{i}}(t)=\left[{x}_{1{A}_{i} }(t), {x}_{2{A}_{i} }(t),\dots ,{x}_{n{A}_{i} }(t)\right],$$

where $n=1, 2,..., N$ VS or clinical variables.

We also calculated the velocity ${\overrightarrow{v}}_{{A}_{i}}$ and acceleration ${\overrightarrow{a}}_{{A}_{i}}$ vectors at each instant of time $t$ as follows:

$${\overrightarrow{v}}_{{A}_{i}}\left(t\right)=\frac{\Delta \left({\overrightarrow{r}}_{{A}_{i}}\left(t\right)\right)}{\Delta t}=\frac{{\overrightarrow{r}}_{{A}_{i}}\left(t\right)-{\overrightarrow{r}}_{{A}_{i}}\left(t-1\right)}{t-\left(t-1\right)}={\overrightarrow{r}}_{{A}_{i}}\left(t\right)-{\overrightarrow{r}}_{{A}_{i}}\left(t-1\right)$$

$${\overrightarrow{a}}_{{A}_{i}}\left(t\right)=\frac{\Delta \left({\overrightarrow{v}}_{{A}_{i}}\left(t\right)\right)}{\Delta t}=\frac{{\overrightarrow{v}}_{{A}_{i}}\left(t\right)-{\overrightarrow{v}}_{{A}_{i}}\left(t-1\right)}{t-\left(t-1\right)}={\overrightarrow{v}}_{{A}_{i}}\left(t\right)-{\overrightarrow{v}}_{{A}_{i}}\left(t-1\right)$$

#### Sepsis position, velocity, and acceleration

Defining sepsis and identifying its onset is a challenging task, and the criteria or methodology adopted can vary. There are several criteria available like SIRS criteria [29], sepsis-3 criteria [5, 30], Angus methodology [31], and Martin methodology [32], among others. The database adopted in this work uses the sepsis-3 criteria to set the sepsis onset; however, the criteria used by the database are not relevant to our approach but just the indication of sepsis onset by some criterion.

Each patient has a sepsis onset with their correspondent values of VS variables. In a simple and immediate configuration, the sepsis position point or just sepsis position would be a unique and static point in the *n*-dimensional space defined by some simple statistical calculation, common for all patients. In a more sophisticated configuration, the sepsis position could be a moving point according to some statistical and/or medical rule of position changing, also applied to all patients as a common sepsis point path. Another issue is that the patients can be grouped by characteristics like gender, age range, comorbidity, among others. Here, the definition of the sepsis position would be made for each group.

In this work, we decided to non-group the patients and use a statistic median to calculate a unique static sepsis position for all patients, as an initial configuration to test our approach, because our goal is to verify the better accuracy between the VS and KF models.

Therefore, considering that ${t}_{s}$ represents the sepsis onset time for the positive patients, we established that the sepsis position vector for all patients were defined by the median values of each VS on the sepsis onset. That is, ${\overrightarrow{r}}_{{B}_{i}}(t)$= ${\overrightarrow{r}}_{B}(t) ={\overrightarrow{r}}_{B}$ will be given by

$${\overrightarrow{r}}_{B}=\left[median\left({x}_{1{A}_{i}}({t}_{s})\right), \dots , median\left({x}_{n{A}_{i}}({t}_{s})\right)\right]i=1, 2, ..., I,$$

where $I$ is the number of positive patients.

Once the vector ${\overrightarrow{r}}_{B}$ has been identified, it will be used to determine the relative position of patients to the sepsis position, at each instant, for positive and negative patients.

#### Movement of patient ${A}_{i}$ relative to sepsis point $B$

As the components of ${\overrightarrow{r}}_{B}$ are composed by the median of VS values at ${t}_{s}$, it is not probable that patient ${A}_{i}$ will reach this exact sepsis position. Thus, after some time, a positive patient ${A}_{i}$ reaches some position (given by ${\overrightarrow{r}}_{{A}_{i}}$) “near” the sepsis position given by ${\overrightarrow{r}}_{B}$, while a negative patient “never” gets “near” this position. There is no special need to calculate in advance “how near” or how the patients’ positions spread around the sepsis position point because the learning process of the prediction model will consider it internally. Therefore, we focused on the relative position vectors ${\overrightarrow{r}}_{{A}_{i}/B}$ of the patients relative to the sepsis position at each instant $t$:

$${\overrightarrow{r}}_{{{A}_{i}}{/B}}\left(t\right)={\overrightarrow{r}}_{{A}_{i}}\left(t\right)-{\overrightarrow{r}}_{B}$$

**Fig. 1:**
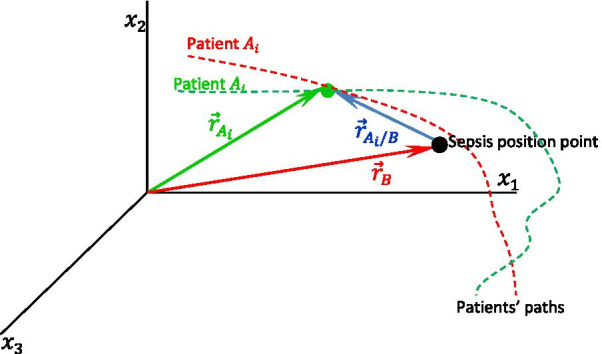
Schematic of ${\overrightarrow{r}}_{{A}_{i}/B}$: the position vector ${\overrightarrow{r}}_{A}$ of patient ${A}_{i}$ relative to the sepsis position vector ${\overrightarrow{r}}_{B}$. Adapted from [27, 28]

Figure 1 shows an example of vectors ${\overrightarrow{r}}_{{A}_{i}}$, ${\overrightarrow{r}}_{B}$, and ${\overrightarrow{r}}_{{A}_{i}/B}$, considering only three VS (3D space). The schematic illustrates the movements of a patient (red) that reached a position “near” the sepsis position point and another patient (green) that did not.

The distance between patient position point and sepsis position point at each instant $t$ is the intensity of position vectors ${\overrightarrow{\mathrm{r}}}_{{\mathrm{A}}_{\mathrm{i}}/\mathrm{B}}$ at each instant $\mathrm{t}$, $\left|{\overrightarrow{r}}_{{A}_{i}/B}(t)\right|$, and it is calculated using the Euclidian Distance formula:

$$\left|{\overrightarrow{r}}_{{A}_{i}/B}(t)\right|= \sqrt{{\left({{r}_{{A}_{i}/B}}_{1}(t)\right)}^{2}+ {\left({{r}_{{A}_{i}/B}}_{2}(t)\right)}^{2}+\dots {\left({{r}_{{A}_{i}/B}}_{n}(t)\right)}^{2}}$$

The velocity vector of patient ${A}_{i}$ relative to the sepsis position vector $B$ is denoted by ${\overrightarrow{v}}_{{A}_{i}/B}$:

$${\overrightarrow{v}}_{{A}_{i}/B}\left(t\right)=\frac{\Delta \left({\overrightarrow{r}}_{{A}_{i}/B}\left(t\right)\right)}{\Delta t}={\overrightarrow{r}}_{{A}_{i}/B}\left(t\right)-{\overrightarrow{r}}_{{A}_{i}/B}\left(t-1\right)$$

Similarly, the acceleration vector is denoted by ${\overrightarrow{a}}_{{A}_{i}/B}$:

$${\overrightarrow{a}}_{{A}_{i}/B}\left(t\right)=\frac{\Delta \left({\overrightarrow{v}}_{{A}_{i}/B}\left(t\right)\right)}{\Delta t}={\overrightarrow{v}}_{{A}_{i}/B}\left(t\right)-{\overrightarrow{v}}_{{A}_{i}/B}\left(t-1\right)$$

#### Projection of vectors ${\overrightarrow{v}}_{{A}_{i}/B}$ and ${\overrightarrow{a}}_{{A}_{i}/B}$ in direction of the vector ${\overrightarrow{r}}_{{A}_{i}/B}$

We considered that the relative movement between a patient and the sepsis point must be “rectilinearized,” that is, it must be viewed at each instant as a potential rectilinear movement of the patient directly to the sepsis point. Using the projection of the vectors ${\overrightarrow{v}}_{{A}_{i}/B}$ and ${\overrightarrow{a}}_{{A}_{i}/B}$ in the direction of the vector ${\overrightarrow{r}}_{{A}_{i}/B}$ is a way to express this “tendency” of patient ${A}_{i}$ to move straight through the sepsis point $B$.

These projections comprise calculating their scalar products with the unit relative position vector ${\overrightarrow{e}}_{i}$, which has the same direction and sense of the relative position vector ${\overrightarrow{r}}_{{A}_{i}/B}$:

$${\overrightarrow{e}}_{i}(t)=\frac{{\overrightarrow{r}}_{{A}_{i}/B}(t)}{\left|{\overrightarrow{r}}_{{A}_{i}/B}(t)\right|}$$

$${\mathrm{Proj}}_{{\overrightarrow{e}}_{i}(t)}^{{\overrightarrow{v}}_{{A}_{i}/B}(t)}={\overrightarrow{v}}_{{A}_{i}/B}(t)\bullet {\overrightarrow{e}}_{i}(t)$$

$${\mathrm{Proj}}_{{\overrightarrow{e}}_{i}(t)}^{{\overrightarrow{a}}_{{A}_{i}/B}(t)}={\overrightarrow{a}}_{{A}_{i}/B}(t)\bullet {\overrightarrow{e}}_{i}(t)$$

The projection of the velocity vector, ${\mathrm{Proj}}_{{\overrightarrow{e}}_{i}(t)}^{{\overrightarrow{v}}_{{A}_{i}/B}(t)},$ is a scalar that can be positive, null, or negative. Its value, at every instant, either maintains, increases, or decreases the distance between the patient ${A}_{i}$ and the sepsis point $B$. Likewise, the projection of the acceleration vector, ${\mathrm{Proj}}_{{\overrightarrow{e}}_{i}(t)}^{{\overrightarrow{a}}_{{A}_{i}/B}(t)},$ is a scalar value that, at every instant, signifies the changes in the rate that patient ${A}_{i}$ and sepsis point $B$ are distancing from or approaching each other.

#### Sepsis early detection and kinematics features

In our study, we propose to calculate the patients’${\overrightarrow{e}}_{i}$, ${\mathrm{Proj}}_{{\overrightarrow{e}}_{i}(t)}^{{\overrightarrow{v}}_{{A}_{i}/B}(t)}$ and ${\mathrm{Proj}}_{{\overrightarrow{e}}_{i}(t)}^{{\overrightarrow{a}}_{{A}_{i}/B}(t)}$ at each instant, and the hypothesis we seek to verify is whether accuracy is gained by including these KF as the input of neural network predictors/classifiers, instead of using VS alone.

If the KF show that a patient, as an *n*-dimensional space point, is approaching the sepsis position with a certain velocity and acceleration during the monitoring time, it could show more precisely that the patient will be diagnosed with sepsis in the future.

Hence, we consider a general prediction/classification mathematical function:

$$f\left({a}_{1}, {a}_{2},..,{a}_{\mathrm{n }}\right)={c}_{1}{a}_{1}+{c}_{2}{a}_{2}+\dots +{c}_{\mathrm{n}}{a}_{\mathrm{n}},$$

where ${a}_{j}$ ($j=1, 2,\dots ,\mathrm{ n}$) are the input parameters and ${c}_{j}$ ($j=1, 2, \dots ,\mathrm{ n}$) are the coefficients to achieve the best accuracy.

Our goal is to compare the resultant of this function using the KF as input parameters with the resultant considering only VS as the input parameters. If we demonstrate that KF increases the accuracy of early detection of sepsis, we will have contributed to our intended objectives.

### Data source and preprocessing

As a data source, we used the clinical multivariate time-series database published in the Early Prediction of Sepsis From Clinical Data: The PhysioNet/Computing in Cardiology Challenge 2019 [33–35]. This database has 40 hourly clinical variables, including VS, laboratory tests, and static patient descriptions, collected from United States hospitals—namely Beth Israel Deaconess Medical Center and Emory University Hospital with respective institutional approval. The authors of the database used the sepsis-3 clinical criteria [5] to define the sepsis onset and included a variable to show the instant it occurs. Only the positive patients have this sign, thus we relied on it to define a precise sepsis position vector and separate the positive and the negative patients. According to what we posed in the kinematics analysis section, we assumed that the last instant of a positive patient series is the sepsis onset time, therefore all the registers after this time were rejected.

The dataset posted publicly for download has 40,336 patients, divided into test sets A and B, both with the 40 hourly clinical variables. From these clinical variables, we selected six common VS monitored in hospitals (Table 1).

**Table 1:** VS selected. Adapted from [33]

#	Measurement	Description	Unit
1	HR	Heart rate	beats/min
2	O_2_Sat	Pulse oximetry	%
3	Temp	Temperature	°C
4	SBP	Systolic blood pressure	mm Hg
5	DBP	Diastolic blood pressure	mm Hg
6	Resp	Respiration rate	breaths/min

The authors of the database challenged contestants to develop algorithms for the early prediction of sepsis and published the results on the corresponding website [35], but the authors plan to publish a paper about the results as soon as possible [36]. The best-ranked result achieved 82.8% accuracy on test set A and 88.8% on test set B using all 40 clinical variables. Because we used only six common VS, it is important to have a point of reference.

**Fig. 2:**
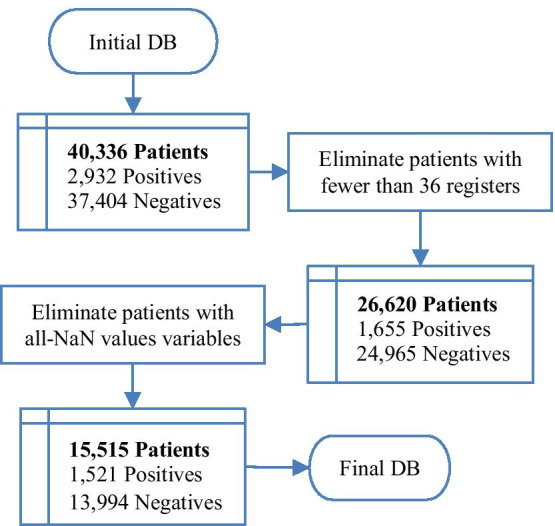
Preprocessing: exclusion flowchart

Because the authors of the database intentionally preserved the missing data and erroneous values, we preprocessed the selected data before setting the sepsis position and calculating the KF. First, it was necessary to define a minimum number of registers, which we could deal with. Once in this work we considered patients who developed sepsis in the ICU, any number of registers we chose would be compatible with the database used because it is sourced from ICU patients. We chose to eliminate patients with fewer than 36 registers, which correspond to three 12-h medical shifts of measured data. Additionally, if a patient had at least one of the six VS with all Not a Number (NaN) values, we eliminated that patient. Figure 2 shows the exclusion flowchart where the number of patients reduced from 40,336 to 15,515.

**Fig. 3:**
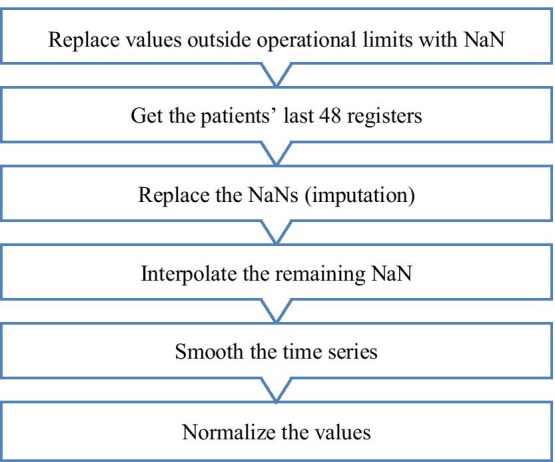
Preprocessing

After the exclusion process, we found VS with absurd values, probably because of sensor reading or typing errors. Here, we set the operating limits for each variable based on [11], and we replaced those values with NaNs. Figure 3 illustrates this step and the subsequent preprocessing steps.

The number of registers varied from patient to patient. Some of them reached hundreds of hours before the instant of sepsis detection whereas others reached sepsis position in a few hours. However, because we intended to use the data as input for neural networks, we established that all the patients should have the same number of registers. Therefore, we arbitrated that the last 48 h (four medical shifts) were "adequate and sufficient" to analyze the data behavior and to determine the risk of sepsis occurrence. For patients who had less than 48 registers, with or without NaNs in their time series, we filled the "previously missing data" with NaNs.

To tackle the NaNs, we used the mean value of each VS of all patients at each hour and proceeded with the imputation of values accordingly. After this process, we linearly interpolated the remaining missing values after verifying that just 1 or 2 h remained with NaN in some VS.

After the imputation, the VS curves showed a ruggedness even bigger. Although the necessity of smoothing the curves was not imperative, we preferred to do this using the moving average of the 3 last values from hour minus 2 to the present hour.

Then, to address the problem of having multiple variable units, we normalized the VS values based on their maximum values. Thus, we fixed the minimum as zero and applied the formula, ${xNorm}_{j}= {x}_{j}/{x}_{j}Max$.

After all these preprocessing procedures, we could calculate the sepsis position components and the KF using the 1521 positive and 13,994 negative patients. With the six VS along with their KF, each patient had 48 registers. Tables 2 and 3 present part of the sample database available in [26], where the six columns in Table 2 are the normalized VS, and the eight columns in Table 3 are the KF, that is, the six components of the unit vector ${\overrightarrow{e}}_{i}$ followed by the scalars ${\mathrm{Proj}}_{{\overrightarrow{e}}_{i}(t)}^{{\overrightarrow{v}}_{{A}_{i}/B}(t)}$ and ${\mathrm{Proj}}_{{\overrightarrow{e}}_{i}(t)}^{{\overrightarrow{a}}_{{A}_{i}/B}(t)}$.

**Table 2:** Sample data with the normalized VS values (ranged from 0 to 1)

HR	O_2_Sat	Temp	SBP	DBP	Resp
0.5582	0.9900	0.9282	0.5486	0.4887	0.3924
0.5677	0.9825	0.9210	0.5570	0.4932	0.4349
0.5779	0.9825	0.9223	0.5742	0.5135	0.4714

**Table 3:** Sample data with the KF values (ranged from − 1 to 1)

e.HR	e.O_2_Sat	e.Temp	e.SBP	e.DBP	e.Resp	${\mathrm{Proj}}_{{\overrightarrow{e}}_{i}(t)}^{{\overrightarrow{v}}_{{A}_{i}/B}(t)}$	${\mathrm{Proj}}_{{\overrightarrow{e}}_{i}(t)}^{{\overrightarrow{a}}_{{A}_{i}/B}(t)}$
0.7251	0.1787	0.0129	0.5146	0.3997	0.1326	0.0217	− 0.0100
0.7231	0.1686	0.0272	0.5131	0.3985	0.1609	0.0012	− 0.0207
0.7185	0.1698	0.0274	0.5167	0.4013	0.1620	− 0.0025	− 0.0037

Moreover, we randomly selected 3316 negative patients from the total 13,994 to balance the number of positive and negative patients according to the processing capacity of the technological infrastructure used. Thus, the final database contained one CSV file with 1521 positive and another CSV file with 3316 negative patients. We labeled the positives with 1 and the negatives with 0 for inputting to the prediction models. Table 4 shows unnormalized values of the basic statistics of the final database.

**Table 4:** Basic statistics of the final database

Variable	Unit	Positive	Negative
Number	#	1521	3316
Age	years	62.35 $\pm$ 16.40	64.06 $\pm$ 15.48
*Gender*	%		
Female		40.28	41.34
Male		59.72	58.66
*Vital signs*			
HR	beats/min	88.05 $\pm$ 16.24	85.65 $\pm$ 14.19
O2Sat	%	97.26 $\pm$ 2.26	97.39 $\pm$ 1.95
Temp	°C	37.13 $\pm$ 0.52	37.10 $\pm$ 0.49
SBP	mm Hg	104.68 $\pm$ 19.88	120.87 $\pm$ 17.24
DBP	mm Hg	62.15 $\pm$ 10.73	60.26 $\pm$ 9.61
Resp	breaths/min	20.78 $\pm$ 4.66	18.40 $\pm$ 4.14

Plus–minus values are means $\pm$ STD. All values are unnormalized

Preprocessing the data is a necessary step in the statistical analysis that can artificially affect the outcome of modeling studies. However, as our approach aims to compare the accuracy of the VS and KF models, all effects from any preprocessing will not compromise the result achieved because they will equally improve or degrade the quality of both models.

### Neural networks for early detection of sepsis

In our research, we calculated KF values using six patients’ VS values at each instant of time. Then, we used these KF values as the input of some neural networks (NN) for the early detection of sepsis.

We decided to use LSTM NN to test our hypothesis because of its “remember” or “forget” features (as it receives inputs sequentially along with the training). These features improve the LSTM’s capacity to discover dependencies in time-series data [37].

LSTM is a variation of a recurrent neural network (RNN) proposed by Hochreiter et al. [38]. The authors solved two known problems of traditional RNNs when dealing with time-series data: (1) the backward propagation error over time becomes extremely high or extremely low, and (2) the progress of the back-propagated error highly depends on the NN weight values. These can cause the weights to vary abnormally, which can increase the time for the learning process significantly [39].

Therefore, we defined an LSTM NN model with the input formed by the VS, and another LSTM model with KF as input. The output layer is always binary (0–negative for sepsis and 1–positive for sepsis). Then, we cross-validated the main LSTM parameters to find those that resulted in better accuracy and lower standard deviation (STD). Thus, we arrived at a model with 128 nodes, 20% dropout, a batch size of 64, and a sigmoid output activation function that was compiled with a binary cross-entropy and Adagrad optimizer [40].

Even though the LSTM NN is sufficient to show if the KF model had a positive impact on accuracy, we compared its results with other models. Hence, we also used a CNN, a linear NN (LNN), and the non-NN classifiers, logistic regression, and decision tree. All of them have the same input and output as the LSTM NN.

Although CNNs are usually applied to visual recognition and text tasks, some researchers have proposed them to classify time-series data [41–43]. Thus, after using the cross-validation process to find the best parameters, we created a CNN model with 64 filters and a kernel size of 3 and used a rectified linear unit (ReLU) as the activation function [44]. The CNN was also compiled with binary cross-entropy and the Adagrad optimizer.

The LNN and the non-NN classifiers do not have characteristics tailored to deal with time-series data. Therefore, we used them only as references, keeping their default parameters.

## Results

Rather than using traditional prediction models, we applied classification models that could discriminate if a set of time-series data represents a positive or negative patient for sepsis at a certain number of hours before sepsis onset (HBS). HBS is the number of hours before the patient's last timestamp; therefore, it represents the earliness of the prediction and affects the number of rows in the model input. In our work, we used 6 HBS; thus, we used only the first 42 rows out of the total of 48 rows available. Hence, when we used VS, the input model had 6 columns and 42 rows, and when we used KF, the input model had 8 columns and 42 rows. In both cases, when the model classifies patients as positive or as negative with certain accuracy, it is predicting sepsis six hours before its onset with the accuracy achieved.

Because the model with KF had a higher-dimensional input, we had to use some model-selection criteria for a fair comparison of the models. After searching for criteria suitable for neural network models, we used the mean accuracies of k-fold cross-validation and the standard deviation. We used the same sepsis position to calculate the KF for the entire database; thus, the data in each fold of the cross-validation process had the same reference.

**Fig. 4:**
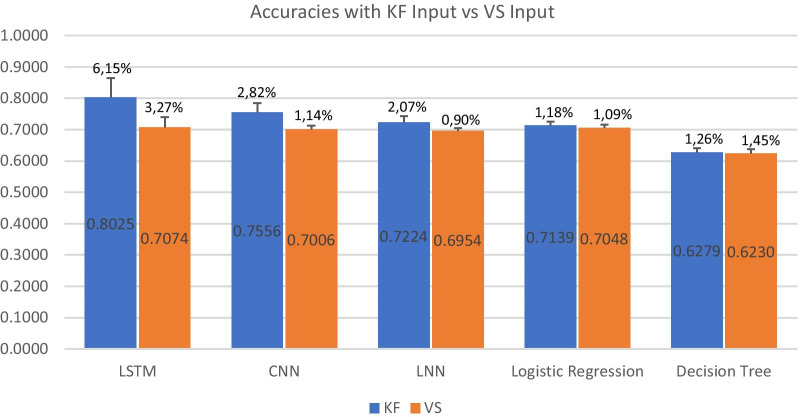
Accuracies of the classifiers comparing KF and VS models. Error bars denote the STDs of the accuracies of the cross-validations

Figure 4 shows the results of the cross-validation process for each model. Inside the bars are the mean accuracies values with KF or with VS as input, that is, the prediction accuracies at 6 HBS.

As required in our hypothesis, we delineated the differences between the accuracies of the KF and VS models: the LSTM model using KF as input achieved a significant difference of 9 compared with the model using VS as input. The CNN models achieved lower accuracies, but the results were similar because using KF as input increased the accuracy by 6 points. As expected, when we tried to classify the patients using an LNN model, the accuracies were particularly lower than the LSTM model, but there was only a slight difference between the KF and VS results. The logistic regression and decision tree classifiers showed virtually no differences between the KF and VS models; moreover, the decision tree reached the worse accuracies of all.

Table 5 presents a comparative summary of the results of each classifier ordered by KF accuracy. The accuracies shown by the LSTM and CNN classifiers were higher. Unlike the other classifiers, they have characteristics that are essential for classifying the data we had constructed: the KF is time-dependent, has an onset (sepsis position), and each register has values that are relative to the onset.

**Table 5:** Comparative summary

#	Classifier	Mean Acc. KF model	Mean Acc. VS model	Mean Diff	STD KF model (%)	STD VS model (%)
1	LSTM	0.8025	0.7074	0.09	6.15	3.27
2	CNN	0.7556	0.7006	0.06	2.82	1.14
3	Linear NN	0.7224	0.6954	0.02	2.07	0.90
4	Logist. Regres	0.7139	0.7048	0.00	1.18	1.09
5	Decision Tree	0.6279	0.6230	0.01	1.26	1.45

**Fig. 5:**
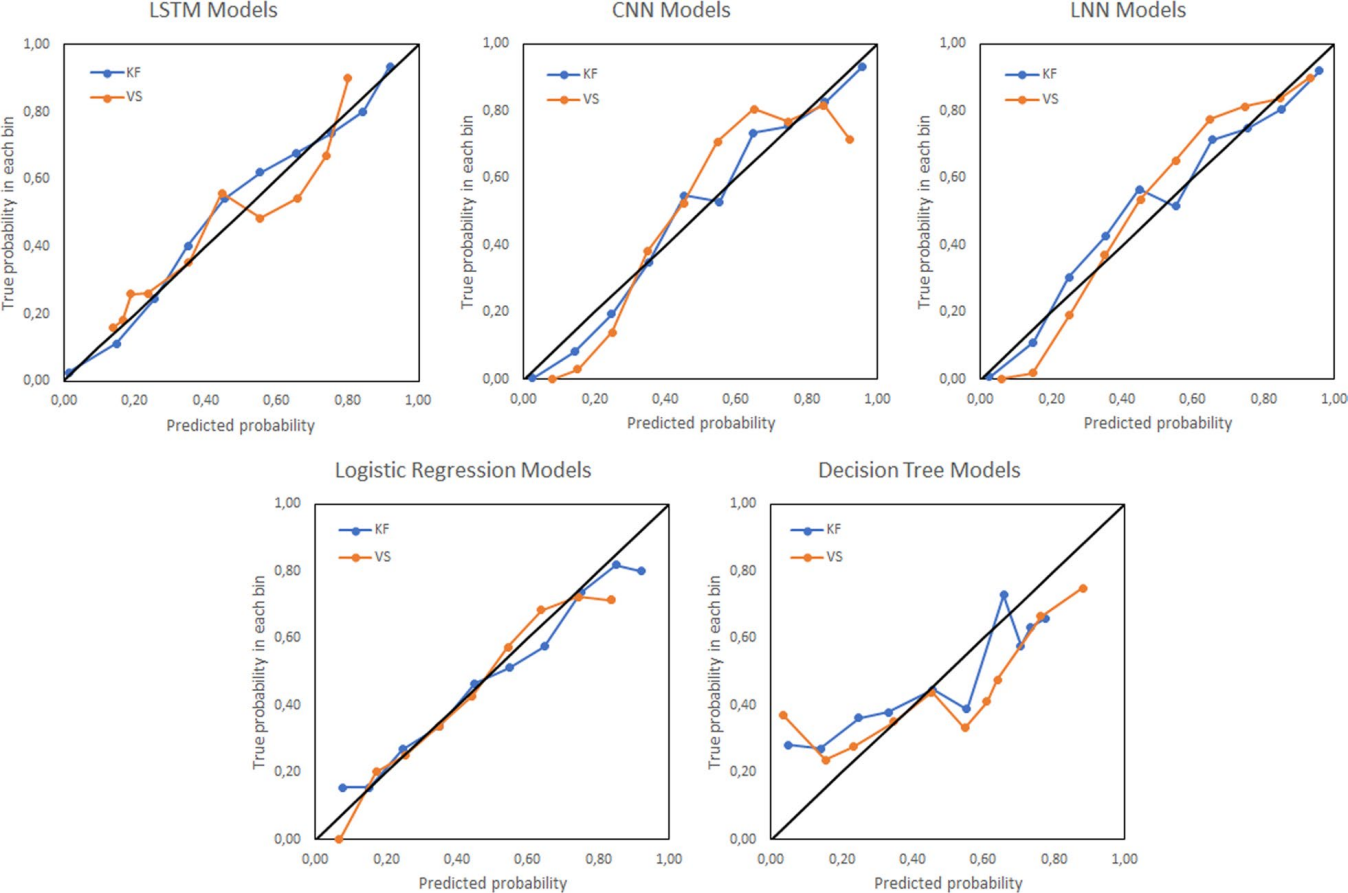
Calibration curves of considered models

Figure 5 shows the calibration curves of the models. As expected, decision tree models are the less calibrated ones.

Our hypothesis became more distinct with the LSTM and CNN classifiers because the differences between the mean accuracies with and without KF as input (Mean Diff. column) were at least three times higher than the mean differences of the other classifiers. As a result, the other classifiers could not achieve our goal, especially if we consider the STDs.

## Discussion

In our approach, we showed a novel way to handle the input parameters of NN-based sepsis classification models to improve their accuracies. Instead of inputting directly the patients’ time-series variables, as is commonly proposed, we treated them as points with velocity and acceleration relative to a sepsis reference and generated a new type of input: the patients’ time-series kinematic features.

KF models have proven to be more accurate than the VS models, because of the higher capability of KF to distinguish positive patients from negative patients, as the distance from the sepsis point is considered at every instant and the speed with which the patients reach the sepsis point.

In the approach we posit, any set of time-series variables can be used as inputs, although in this work, we selected only six VS commonly monitored in hospitals: heart rate (HR), pulse oximetry (O_2_Sat), temperature (Temp), systolic blood pressure (SBP), diastolic blood pressure (DBP), respiration rate (Resp). Therefore, this work can be easily reproduced and expanded by incorporating data from additional and different databases.

In this work, we also assumed that the last 48 h of VS data were a sufficient window size to prove our hypothesis. However, future works can verify if different window sizes affect the results.

Likewise, the criteria for selecting the VS variables can be improved by fitting probabilistic models applied to their time-series values or by any new technique for selecting the most statistically significant time-series variable for prediction, such as the one proposed by [13]. The entropy of the VS dynamics [20, 23–25] can also be useful in this matter.

In addition, other missing data imputation, interpolation, and normalization procedures certainly would contribute to the preprocessing stage and overall results. It would also be useful to verify how other options of preprocessing methods affect the models’ accuracies with KF and VS as input.

The proposed kinematics approach can also be applied in real-time vital sign monitoring. Here, the position of the sepsis point must be previously defined using the existing data of positive patients, and each new positive patient datum can be used to recalculate a new sepsis position. In this manner, all KF can be calculated in real-time and imputed in the NN model at every instant.

**Fig. 6:**
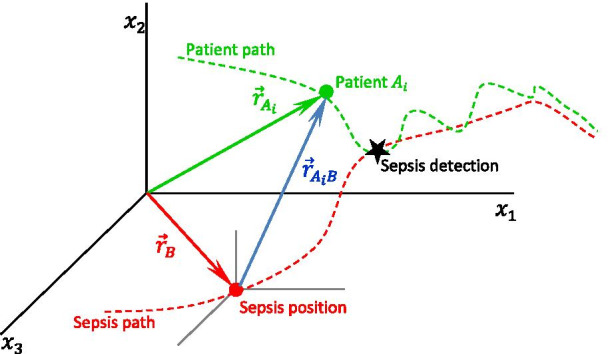
Sepsis path of a moving sepsis position

Although the assumption of a static median for the sepsis position point is a simplified way used as baseline to test our hypothesis, there are different ways of defining the sepsis position point. It can be a moving point set according to some statistical method of changing variable values or according to some clinical rules relative to the variables (Fig. 6). Additionally, it can be set combined with clustering techniques to find a moving sepsis position point for each group of patients, depending on their features, such as previous diseases, gender, age range, ethnic group. Therefore, other ways to define sepsis position point will be an enhancement for this work and further validation of the approach proposed here.

Among the six components of the sepsis position vector, only two of them are included as variables in the qSOFA score, which, together with the SOFA score, is part of the sepsis-3 criteria to set the sepsis onset. In this work, the “unnormalized” values of components SBP and Resp achieved 90.91 and 21.65 respectively, which mean the attendance of 2-point of the qSOFA score ($SBP \le 100, Resp \ge 22$), which is sufficient to define a positive condition for sepsis. However, this is not true all the time because the positiveness for sepsis could come from the third qSOFA score variable. In any case, our approach does not depend on this verification, as different variables can be chosen independently of the sepsis criteria variables.

We identified the KF model of the LSTM classifier achieved the highest accuracy. To compare its performance with other works that used VS as input for early detection of sepsis, we present Table 6 that shows their HBS and area under the receiver operating characteristic (AUROC) values. The KF model reached a similar AUROC but a better HBS value, excluding the “outlier” result achieved by Mohammed et al. [15].

**Table 6:** AUROC of LSTM KF model compared with other works

#	Work	Model	HBS	AUROC
1	Lauritsen et al. [19]	CNN-LSTM	03	0.856
2	Mao et al. [12]	Gradient tree boosting	04	0.850
3	KF model	LSTM	06	0.835
4	Kamaleswaran et al. [14]	CNN	02–08	0.830
5	Mohammed et al. [15]	Support vector machine	17.4	0.781

Despite proving our hypothesis using ordinary classifiers, we pondered the possibility of improving the KF model performance using new neural network architectures, based on CNN and LSTM [16–19], or new methodologies/algorithms [9, 12].

Furthermore, we think that it is essential to compare our results with the results obtained using other databases to reveal any bias, even though it is difficult to locate public databases that identify the disease onset.

## Conclusion

In recent years, researchers have proposed various models for predicting patient illnesses, seeking to use fewer variables while maximizing accuracy and speed of prediction. Applying our new and innovative kinematics approach for early detection of sepsis using NN classifiers will prove to be a valuable and more accurate approach than considering only simple VS input variables, showing its significant potential in the development of this scientific knowledge.

## Data Availability

The sample data and essential source code are available at http://www.github.com/marciofreire/KANNEDS.

## Abbreviations

CNN: Convolutional neural network
HBS: Hours before onset
KANNEDS: Kinematics approach with neural networks for early detection of sepsis
KF: Kinematic feature
LSTM: Long short-term memory
NaN: Not a Number
NN: Neural network
STD: Standard deviation
VS: Vital sign

## References

[1] Singer M, Deutschman CS, Seymour CW, Shankar-Hari M, Annane D, Bauer M (2016). The Third International Consensus Definitions for Sepsis and Septic Shock (Sepsis-3). JAMA.

[2] Seymour CW, Liu VX, Iwashyna TJ, Brunkhorst FM, Rea TD, Scherag A (2016). Assessment of clinical criteria for sepsis for the third international consensus definitions for sepsis and septic shock (sepsis-3). JAMA J Am Med Assoc.

[3] Fleischmann C, Scherag A, Adhikari NKJ, Hartog CS, Tsaganos T, Schlattmann P (2016). Assessment of global incidence and mortality of hospital-treated sepsis current estimates and limitations. Am J Respir Crit Care Med.

[4] Giamarellos-Bourboulis EJ, Tsaganos T, Tsangaris I, Lada M, Routsi C, Sinapidis D (2017). Validation of the new Sepsis-3 definitions: proposal for improvement in early risk identification. Clin Microbiol Infect.

[5] Rhodes A, Evans LE, Alhazzani W, Levy MM, Antonelli M, Ferrer R (2016). Surviving sepsis campaign: international guidelines for management of sepsis and septic Shock. Crit Care Med.

[6] Song JU, Sin CK, Park HK, Shim SR, Lee J (2018). Performance of the quick Sequential (sepsis-related) Organ Failure Assessment score as a prognostic tool in infected patients outside the intensive care unit: a systematic review and meta-analysis. Crit Care.

[7] Rousselot J, Decotignie J-D. Wireless communication systems for continuous multiparameter health monitoring. In: IEEE International conference on ultra-wideband. Vancouver: IEEE; 2009. p. 480–4.

[8] Cruz MF, Cavalcante CAMT, Sá Barretto ST (2018). Using OPC and HL7 standards to incorporate an industrial Big Data historian in a health IT environment. J Med Syst.

[9] Henry KE, Hager DN, Pronovost PJ, Saria S (2015). A targeted real-time early warning score (TREWScore) for septic shock. Sci Transl Med.

[10] Manaktala S, Claypool SR (2017). Evaluating the impact of a computerized surveillance algorithm and decision support system on sepsis mortality. J Am Med Inform Assoc.

[11] Horng S, Sontag DA, Halpern Y, Jernite Y, Shapiro NI, Nathanson LA (2017). Creating an automated trigger for sepsis clinical decision support at emergency department triage using machine learning. PLoS ONE.

[12] Mao Q, Jay M, Hoffman JL, Calvert J, Barton C, Shimabukuro D (2018). Multicentre validation of a sepsis prediction algorithm using only vital sign data in the emergency department, general ward and ICU. BMJ Open.

[13] Bock C, Gumbsch T, Moor M, Rieck B, Roqueiro D, Borgwardt K (2018). Association mapping in biomedical time series via statistically significant shapelet mining. Bioinformatics.

[14] Kamaleswaran R, Akbilgic O, Hallman MA, West AN, Davis RL, Shah SH (2018). Applying artificial intelligence to identify physiomarkers predicting severe sepsis in the PICU. Pediatr Crit Care Med.

[15] Mohammed A, Van Wyk F, Chinthala LK, Khojandi A, Davis RL, Coopersmith CM, et al. Temporal differential expression of physiomarkers predicts sepsis in critically ill adults. Shock. 2020 Sep 28; Publish Ah. Available from 10.1097/SHK.0000000000001670. 10.1097/SHK.0000000000001670 PMC8352046 32991797

[16] Perng J-W, Kao I-H, Kung C-T, Hung S-C, Lai Y-H, Su C-M. Mortality prediction of septic patients in the emergency department based on machine learning. J Clin Med. 2019 Nov 7 [cited 2021 Jan 27];8(11):1906. Available from https://www.mdpi.com/2077-0383/8/11/1906. 10.3390/jcm8111906 PMC6912277 31703390

[17] Li X, André Ng G, Schlindwein F. Convolutional and recurrent neural networks for early detection of sepsis using hourly physiological data from patients in intensive care unit. In: 2019 Computing in Cardiology Conference (CinC). Computing in Cardiology; 2019.

[18] Lin C, Zhangy Y, Ivy J, Capan M, Arnold R, Huddleston JM, et al. Early diagnosis and prediction of sepsis shock by combining static and dynamic information using convolutional-LSTM. In: Proceedings—2018 IEEE international conference on healthcare informatics, ICHI 2018. Institute of Electrical and Electronics Engineers Inc.; 2018. p. 219–28.

[19] Lauritsen SM, Kalør ME, Kongsgaard EL, Lauritsen KM, Jørgensen MJ, Lange J (2020). Early detection of sepsis utilizing deep learning on electronic health record event sequences. Artif Intell Med.

[20] Lake DE, Richman JS, Griffin MP, Moorman JR (2002). Sample entropy analysis of neonatal heart rate variability. Am J Physiol Integr Comp Physiol.

[21] Pincus SM, Gladstone IM, Ehrenkranz RA. A regularity statistic for medical data analysis. J Clin Monit. 1991 Oct [cited 2021 Jan 27];7(4):335–45. Available from 10.1007/BF01619355. 10.1007/BF01619355 1744678

[22] Richman JS, Moorman JR (2000). Physiological time-series analysis using approximate entropy and sample entropy. Am J Physiol Circ Physiol.

[23] Ahmad S, Ramsay T, Huebsch L, Flanagan S, McDiarmid S, Batkin I (2009). Continuous multi-parameter heart rate variability analysis heralds onset of sepsis in adults. PLoS ONE.

[24] Drewry AM, Fuller BM, Bailey TC, Hotchkiss RS. Body temperature patterns as a predictor of hospital-acquired sepsis in afebrile adult intensive care unit patients: a case-control study. Vol. 17, Critical Care. 2013 [cited 2021 Jan 27]. Available from http://ccforum.com/content/17/5/R200. 10.1186/cc12894 PMC3906745 24028682

[25] Shashikumar SP, Stanley MD, Sadiq I, Li Q, Holder A, Clifford GD (2017). Early sepsis detection in critical care patients using multiscale blood pressure and heart rate dynamics. J Electrocardiol.

[26] Freire Cruz M, Ono N, Huang M, Altaf-Ul-Amin M, Kanaya S, Cavalcante CAMT. Repository of the project KANNEDS—kinematics analysis and neural networks for early detection of sepsis. 2020. Available from http://www.github.com/marciofreire/KANNEDS. 10.1186/s12911-021-01529-3 PMC8138930 34016115

[27] Beer FP, Johnston ER, Mazurek DF, Cornwell PJ, Eisenberg ER (2009). Vector mechanics for engineers: statics and dynamics.

[28] Meriam JL, Kraige LG (2012). Engineering mechanics-dynamics.

[29] Bone RC, Balk RA, Cerra FB, Dellinger RP, Fein AM, Knaus WA (1992). Definitions for sepsis and organ failure and guidelines for the use of innovative therapies in sepsis. Chest.

[30] Levy MM, Evans LE, Rhodes A (2018). The surviving sepsis campaign bundle: 2018 update. Intensive Care Med.

[31] Angus DC, Linde-Zwirble WT, Lidicker J, Clermont G, Carcillo J, Pinsky MR (2001). Epidemiology of severe sepsis in the United States: analysis of incidence, outcome, and associated costs of care. Crit Care Med.

[32] Martin GS, Mannino DM, Eaton S, Moss M. The epidemiology of sepsis in the United States from 1979 through 2000. N Engl J Med. 2003 [cited 2021 Jan 16];16:1546–54. Available from www.nejm.org. 10.1056/NEJMoa022139 12700374

[33] Reyna MA, Josef CS, Jeter R, Shashikumar SP, Westover MB, Nemati S (2020). Early prediction of sepsis from clinical data. Crit Care Med.

[34] Goldberger AL, Amaral LAN, Glass L, Hausdorff JM, Ivanov PC, Mark RG (2000). PhysioBank, PhysioToolkit, and PhysioNet. Circulation.

[35] Reyna M, Josef C, Jeter R, Shashikumar S, Moody B, Westover MB, et al. Early prediction of sepsis from clinical data—the PhysioNet computing in cardiology challenge 2019 (version 1.0.0). 2019. Available from https://physionet.org/content/challenge-2019/1.0.0/. 10.1097/CCM.0000000000004145 PMC6964870 31939789

[36] Reyna M, Clifford G. Voting of predictive models for clinical outcomes: consensus of algorithms for the early prediction of sepsis from clinical data and an analysis of the PhysioNet/CinC Challenge 2019. arXiv:2012.11013v1. 2020.

[37] Fagerström J, Bång M, Wilhelms D, Chew MS (2019). LiSep LSTM: a machine learning algorithm for early detection of septic shock. Sci Rep.

[38] Hochreiter S, Schmidhuber J (1997). Long short-term memory. Neural Comput.

[39] Hochreiter S, Schmidhuber JJ. Bridging long time lags by weight guessing and “Long short term memory.” In: Silva FL, Principe JC, Almeida LB, editors. Spatiotemporal models in biological and artificial systems. IOS Press; 1996. p. 65–72.

[40] Duchi J, Singer Y (2011). Adaptive subgradient methods for online learning and stochastic optimization * Elad Hazan. J Mach Learn Res.

[41] Yi S, Ju J, Yoon M-K, Choi J. Grouped convolutional neural networks for multivariate time series. arXiv:1703.09938. 2017.

[42] Liu CL, Hsaio WH, Tu YC (2019). Time series classification with multivariate convolutional neural network. IEEE Trans Ind Electron.

[43] Yazdanbakhsh O, Dick S. Multivariate time series classification using dilated convolutional neural network. In: 36th International Conference on Machine Learning. Long Beach; 2019.

[44] Nair V, Hinton GE. Rectified linear units improve restricted boltzmann machines. In: The 27a International Conference on Machine Learning. Haifa; 2010.

